# A longitudinal study of SARS-CoV-2-infected patients reveals a high correlation between neutralizing antibodies and COVID-19 severity

**DOI:** 10.1038/s41423-020-00588-2

**Published:** 2021-01-06

**Authors:** Vincent Legros, Solène Denolly, Manon Vogrig, Bertrand Boson, Eglantine Siret, Josselin Rigaill, Sylvie Pillet, Florence Grattard, Sylvie Gonzalo, Paul Verhoeven, Omran Allatif, Philippe Berthelot, Carole Pélissier, Guillaume Thiery, Elisabeth Botelho-Nevers, Guillaume Millet, Jérôme Morel, Stéphane Paul, Thierry Walzer, François-Loïc Cosset, Thomas Bourlet, Bruno Pozzetto

**Affiliations:** 1 CIRI – Centre International de Recherche en Infectiologie, Team EVIR, Univ Lyon, Université Claude Bernard Lyon 1, Inserm, U1111, CNRS, UMR5308, ENS Lyon, 46 allée d’Italie, F-69007 Lyon, France; 2 Université de Lyon, VetAgro Sup, Marcy-l’Étoile, France; 3 Department of Infectious Agents and Hygiene, University-Hospital of Saint-Etienne, Saint-Etienne, France; 4 Department of Immunology, University-Hospital of Saint-Etienne, Saint-Etienne, France; 5 CIRI – Centre International de Recherche en Infectiologie, Team GIMAP, Univ Lyon, Université Claude Bernard Lyon 1, Inserm, U1111, CNRS, UMR5308, ENS Lyon, 46 allée d’Italie, F-69007 Lyon, France; 6 Department of Infectious Diseases, University-Hospital of Saint-Etienne, Saint-Etienne, France; 7 Department of Occupational Medicine, University-Hospital of Saint-Etienne, Saint-Etienne, France; 8 Department of Intensive Care and Resuscitation (Réanimation G), University-Hospital of Saint-Etienne, Saint-Etienne, France; 9 Laboratoire Interuniversitaire de Biologie de la Motricité, Université de Lyon, Université Jean Monnet, Saint-Etienne, France; 10 Department of Anesthesiology and Critical Care, University-Hospital of Saint-Etienne, Saint-Etienne, France

**Keywords:** Antibody, Pathogenesis, Humoral response, COVID-19, SARS-CoV-2, Prognostic markers, Viral infection

## Abstract

Understanding the immune responses elicited by SARS-CoV-2 infection is critical in terms of protection against reinfection and, thus, for public health policy and vaccine development for COVID-19. In this study, using either live SARS-CoV-2 particles or retroviruses pseudotyped with the SARS-CoV-2 S viral surface protein (Spike), we studied the neutralizing antibody (nAb) response in serum samples from a cohort of 140 SARS-CoV-2 qPCR-confirmed infections, including patients with mild symptoms and also more severe forms, including those that required intensive care. We show that nAb titers correlated strongly with disease severity and with anti-spike IgG levels. Indeed, patients from intensive care units exhibited high nAb titers; conversely, patients with milder disease symptoms had heterogeneous nAb titers, and asymptomatic or exclusive outpatient-care patients had no or low nAbs. We found that nAb activity in SARS-CoV-2-infected patients displayed a relatively rapid decline after recovery compared to individuals infected with other coronaviruses. Moreover, we found an absence of cross-neutralization between endemic coronaviruses and SARS-CoV-2, indicating that previous infection by human coronaviruses may not generate protective nAbs against SARS-CoV-2. Finally, we found that the D614G mutation in the spike protein, which has recently been identified as the current major variant in Europe, does not allow neutralization escape. Altogether, our results contribute to our understanding of the immune correlates of SARS-CoV-2-induced disease, and rapid evaluation of the role of the humoral response in the pathogenesis of SARS-CoV-2 is warranted.

## Introduction

Severe acute respiratory syndrome coronavirus 2 (SARS-CoV-2) first emerged in late 2019 in Wuhan, China. According to John Hopkins University and Coronavirus Resource Center, the disease caused by SARS-CoV-2, named coronavirus disease (COVID-19), has caused over 750,000 deaths worldwide, with over 21 million infected individuals, by mid-August 2020, figures that are likely to be underestimated. The hallmark of the disease is acute respiratory distress syndrome, but other nonspecific symptoms such as sore throat, dry cough, fever, fatigue, muscle aches, runny nose, and diarrhea are frequently present.^1^ Neurological disorders have also been reported, with headache, nausea, vomiting, anosmia and ageusia, acute cerebrovascular disease, Guillain–Barré syndrome, and impaired consciousness.^2^

Understanding the immune responses elicited by SARS-CoV-2 infection is critical in terms of protecting against reinfection and, thus, for public health policy and vaccine development. One of the key functions in acquired immune responses is attributed to neutralizing antibodies (nAbs), which are generally associated with virus clearance and protection.^3,4^ Several reports indicate that most individuals recovering from SARS-CoV-2 infection develop IgM, IgG, and IgA responses targeting the nucleocapsid (N) or the spike (S) protein of SARS-CoV-2 virions at 7–14 days after infection.^5–7^ In addition, nAbs have been identified in patients, suggesting that SARS-CoV-2 infection may generate a robust immune response.^7–9^ Considering the lack of perspectives on the immune correlates of protection against SARS-CoV-2, it is tempting to draw conjecture from the immune responses elicited by other human coronaviruses. For example, nAb activity in patients infected with endemic coronaviruses can rapidly wane other time, as reinfection is frequently described;^10^ in contrast, nAbs against SARS-CoV and Middle East respiratory syndrome-related coronavirus can be detected for up to 36 months.^11,12^ It is therefore urgent to evaluate the nAb response elicited by SARS-CoV-2 infection, the factors associated with its robustness, and its persistence.

In this study, nAb activity in serum samples from a cohort of 140 quantitative PCR (qPCR)-confirmed cases of SARS-CoV-2 infection was quantified. We show that nAb titers correlate strongly with disease severity. Importantly, we also quantified the persistence of nAb activity, which indicated a relatively rapid decline in nAbs after recovery. Moreover, we observed an absence of cross-protection conferred by previous infection by endemic coronaviruses. Finally, we found that the D614G mutation in the spike protein, recently identified as the major variant now found in Europe,^13^ did not induce nAb escape.

## Materials and methods

### Ethics

This study was approved by the Ethics Committee of the University Hospital of Saint-Etienne (reference number IRBN512020/CHUSTE).

### Patients and origin of samples

A total of 140 patients followed at the University Hospital of Saint-Etienne were enrolled between March and May 2020. In all patients, nasopharyngeal swabs were obtained, which tested positive for SARS-CoV-2 RNA by reverse transcriptase qPCR (RT-qPCR) assay. The patients were classified into 3 groups according to their medical care: 44 were admitted to the intensive care unit (ICU), 42 were hospitalized (HOS) without receiving care in the ICU, and 54 were given exclusive outpatient care (EOC), including 8 asymptomatic cases (ASYs).

Time post onset was defined as the time after onset of the first symptoms.

For the ICU and HOS groups, 3–4 serum samples were collected at 3 periods of follow-up post onset: 0–15, 16–30, and > 30 days. For the EOC group, 2 serum samples were collected 13–62 days post onset.

### Seroneutralization assay using wild-type SARS-CoV-2

The viral strain (RoBo strain), which was cultured on Vero-E6 cells (ATCC CRL-1586), used for the nAb assay was a clinical isolate obtained from a nasopharyngeal aspirate of a patient HOS at the University Hospital of Saint-Etienne for severe COVID-19. The strain was diluted in Dulbecco’s modified Eagle’s medium–2% fetal calf serum in aliquots containing 100–500 tissue culture infectious doses 50% (TCID_50_) per ml. Each serum specimen was diluted 1 : 10 and serial twofold dilutions were mixed with an equal volume (100 µl each) of virus. After gentle shaking for 30 min at room temperature, 150 µl of the mixture was transferred to 96-well microplates covered with Vero-E6 cells. The plates were then placed at 37 °C in a 5% CO_2_ incubator. Measurements were obtained microscopically 5–6 days later when the cytopathic effect of the virus control reached ~100 TCID_50_/150 µl. The serum was considered to have protected the cells if >50% of the cell layer was preserved. The neutralizing titer is expressed as the inverse of the higher serum dilution that protected the cells.

### SARS-CoV-2 pseudoparticle preparation and neutralization

SARS-CoV-2 spike-pseudotyped murine leukemia virus (MLV) retrovirus particles were produced as we described for SARS-CoV.^14^ Briefly, HEK293T cells (ATCC CRL-1573) were transfected with constructs expressing MLV Gag-Pol, the green fluorescent protein (GFP) reporter, and the SARS-CoV-2 spike (a kind gift from D. Lavillette). A plasmid encoding the spike protein harboring the D614G mutation was generated by PCR mutagenesis. Control pseudoparticles pseudotyped with the unrelated RD114 virus surface glycoprotein (from a cat endogenous virus) or with the vesicular stomatitis virus (VSV) G protein were generated as previously described.^15^ For neutralization assays, a sample of ~1 × 10^3^ pseudoparticles was incubated with a 100-fold dilution of sera or control antibodies for 1 h at 37 °C before infection of Vero-E6R cells. For ID50 (*i.e*., serum dilution that inhibits 50% of the infectivity) determination, serial threefold dilutions of sera from ICU patients were mixed with the pseudoparticles and treated as described above. At 72 h post transduction, the percentage of GFP-positive cells was determined by flow cytometry. As a control, the same procedure was performed using RD114 pseudoparticles.

Anti-spike SARS-CoV-2 RBD (Sino Biological) and anti-gp70 RD114 (ViroMed Biosafety Labs) antibodies were used as positive and negative control nAbs, respectively.

### Antibody-dependent enhancement of SARS-CoV-2pp

For antibody-dependent enhancement (ADE) assays, a sample of 1 × 10^3^ pseudoparticles was incubated with 100-fold and 5000-fold dilutions of sera or control antibodies 1 h at 37 °C before infection of THP-1 cells (ATCC TIB-202). At 72 h post transduction, the percentage of GFP-positive cells was determined by flow cytometry. As a control, the same procedure was performed using VSV-G pseudoparticles.

### Commercial kits for measuring IgG antibodies against SARS-CoV-2

Two commercially available kits were used for measuring anti-SARS-CoV-2 IgG antibodies: the LIAISON® SARS-CoV-2 S1/S2 kit (Diasorin) to measure antibodies against S1-S2 proteins and the ARCHITECT SARS-CoV-2 IgG kit (Abbott Laboratories) to measure antibodies to the viral nucleoprotein.

### Statistical analysis

Statistical analysis was performed using GraphPad Prism-6 software. Significance values were calculated using the Kruskal–Wallis test and Dunn’s multiple comparison test or *t*-test, depending on the groups. Spearman’s coefficient and *p*-value were calculated to evaluate the correlation between variables. Second-order polynomial regression was plotted using lines and ribbons depicting the 95% confidence intervals. *P*-values < 0.05 were considered statistically significant and the following denotations were used: ****P ≤ 0.0001; ***P ≤ 0.001; **P ≤ 0.01; *P ≤ 0.05; ns (not significant), *P* > 0.05.

## Results

### COVID-19 patients and clinical information

A total of 140 consenting patients from the University Hospital of Saint-Etienne (France) with laboratory-confirmed SARS-CoV-2 infections were enrolled in this study. Among them, 44 patients were admitted to the ICU, 42 were HOS without receiving care in the ICU, and 54 were given EOC, including 8 ASY patients. Moreover, nine serum specimens from subjects infected by seasonal coronaviruses were available for the study. A total of 299 blood samples were collected from the patients at various time points post onset of COVID-19 symptoms, as based on the availability of discarded blood samples collected for routine clinical management. Importantly, several patients were sampled at least three times, 19 ICU, 14 HOS, and 4 EOC patients, to up to 117 days post onset.

Overall, this cohort recapitulated the variability of COVID-19 severity and the common comorbidities already identified.^16–18^ A full summary of the patient characteristics is presented in Table 1. The main clinical symptoms in the ICU and HOS groups were fever (90.1%), dyspnea (56.3%), cough (53.5%), asthenia (29.6%), diarrhea (23.9%), myalgia (8.5%), sputum production (7.0%), and anosmia/ageusia (4.2%). There were no differences in clinical symptoms between these two groups, which differed essentially with regard to disease severity. In the EOC group, moderate symptoms were mainly fever, cough, and asthenia.

**Table 1:** Characteristics of the three groups of COVID-19 patients

Characteristics	Patient group		
	Intensive care ICU (*n* = 44)	Hospitalized HOS (*n* = 42)	Ambulatory EOC + ASY (*n* = 54)
M/F sex ratio	3.9	1.6	0.4
Age (years), median (IQR)	70 (64–75.8)	82 (65–85.2)	37 (32–50.5)
Comorbidities (%)			
Age > 70 years	52.3	69.0	7.4
Obesity	25.0	9.7	0
Hypertension	62.5	61.3	0
Diabetes	30.0	22.6	0
Cardiovascular disease	12.5	41.9	0
Kidney failure	12.5	6.4	0
Chronic respiratory diseases	17.5	16.1	0
Malignancy	5	19.4	0
Severity criteria (%)			
Respiratory rate > 30/min	70	6.5	0
Blood oxygen saturation <92%	97.5	22.6	0
Severe injuries by CT scan	92.5	38.7	0
Oxygen and/or mechanical ventilation needs	100 0	45.1	0
Deceased (%)	16.3	15.4	0

### Pseudoparticle neutralization correlates with wild-type SARS-CoV-2 neutralization

In vitro neutralization of live virus is considered the gold standard method for the assessment of nAbs. However, SARS-CoV-2 requires a BSL-3 facility and the assessment is time consuming. Hence, we developed a SARS-CoV-2 pseudoparticle assay, named the SARS-CoV-2pp assay, to quickly, safely and reliably evaluate nAb activity. To identify nonspecific neutralizing activity, each serum examined with the SARS-CoV-2pp assay was tested in parallel with RD114pp (Supplemental Fig. 1), i.e., pseudoparticles coated with the surface glycoprotein of the cat endogenous retrovirus RD114.^15^

**Fig. 1:**
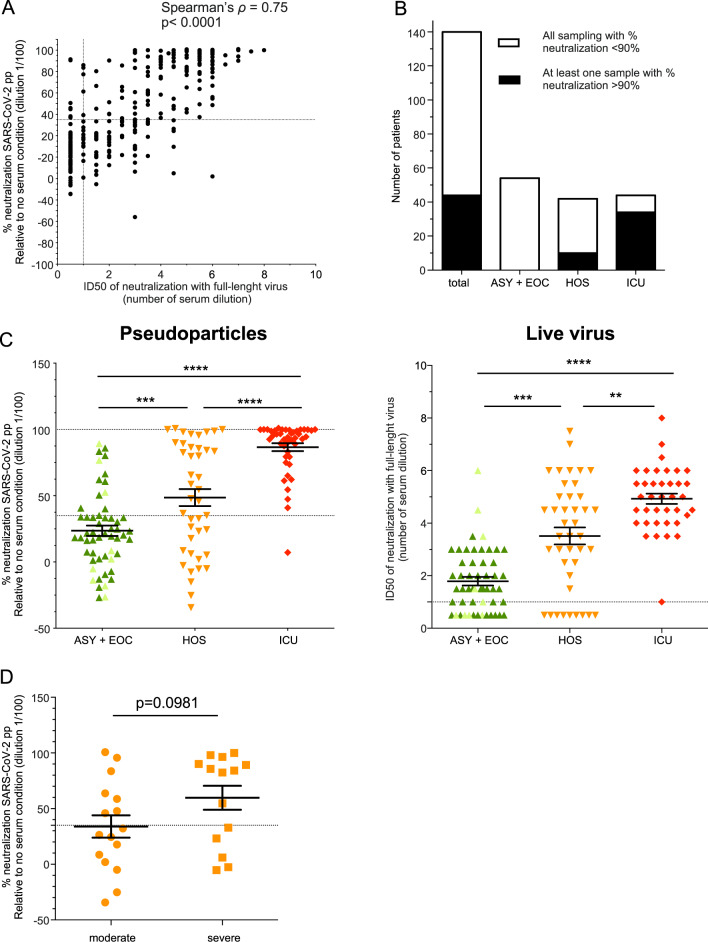
Serum neutralization of SARS-CoV-2 correlates with the hospitalization units of COVID-19 patients. **A** Correlation between the ID50 of live virus, as plotted as number of dilutions (twofold dilutions starting from serum diluted at 1/10) with the percentage of neutralization of SARS-CoV-2pp for all tested samples. **B** Number of patients classified in the indicated groups according to the percentage of neutralization. In white: samples that induced a percentage of neutralization below 90%. **C** Comparison of the percentage of neutralization with SARS-CoV-2pp (left) or ID50 with live virus (right) for each patient classified according to the hospitalization unit. For patients with serial serum samples, the sera collected at the time closest to twenty days post onset of symptoms were chosen. In light green are asymptomatic patients (ASY) among the EOC patients. **D** Percentage of neutralization according to the severity of symptoms in HOS patients. The cutoff for neutralization (35%) was set using the mean neutralization of a 1/100 dilution of negative sera + 2 SD^58^ for SARS-CoV-2pp. For the wild-type virus, the cutoff for the ID50 was set at the 1/10 dilution (first tested dilution), as all negative sera were below this threshold

We first compared the neutralizing activity of the serum samples from COVID-19 patients using the two assays, i.e., live SARS-CoV-2 and pseudoparticles. For live virus, neutralization was assessed by the ID50 (serum dilution that inhibits 50% of the infectivity); for pseudoparticle assays, it was expressed as the percent neutralization at a 1/100 serum dilution relative to a no serum condition. The nAb activity of each serum sample from the 140 patients as well as negative sera, i.e., prepandemic sera, was blindly quantified using either method. Similar results were obtained (Fig. 1A), as indicated by the high Spearman’s rank correlation (*ρ* = 0.75), with <1.5% significantly discordant points. Hence, neutralization assays based on SARS-CoV-2pp can be reliably used to quantify nAb activity.

### The SARS-CoV-2 nAb response correlates with COVID-19 symptom severity

To explore the humoral immune response generated after SARS-CoV-2 infection, we compared the nAb activity of serum samples from patients as a function of the severity of their symptoms using both SARS-CoV-2pp and wild-type virus.

We found that most patients from the different groups produced nAbs, although the neutralizing activity was highly variable (Fig. 1C). Moreover, 44 patients (31.4%) exhibited robust neutralization, allowing over 90% neutralization in the SARS-CoV-2pp assay (Fig. 1B, total).

Strikingly, we found that ICU patients displayed high nAb activity compared to other groups with milder disease symptoms, such as HOS and EOC patients (Fig. 1C). Indeed, only one ICU patient did not develop a nAb response at the time of sampling; for the HOS and EOC patients, nAb activity was lower and more heterogeneous. Specifically, 21.9 and 25% of the patients in HOS and EOC categories, respectively, did not develop nAbs able to neutralize wild-type SARS-CoV-2 at the time of sampling (Fig. 1C right). In addition, the serum from 34.5% of HOS patients had no neutralizing activity against SARS-CoV-2pp and this number increased to 70.7% in the EOC patients (Fig. 1C left).

Concerning HOS patients, we sought to classify them according to the severity of their symptoms. Accordingly, we defined as “severe” patients who had a respiratory rate > 30/min and/or blood oxygen saturation < 92% and/or lung lesions observed by computed tomography scan and/or intensive oxygen therapy. HOS patients were classified as “moderate” if the above criteria were not met. However, we found no significant correlation (*p* = 0.0981) between the severity of symptoms of the HOS patients and their nAb titers, even though neutralization was higher in the serum of those with more severe disease (Fig. 1D). This indicated that the severity of symptoms is not the only factor explaining the diversity of nAb activity among HOS patients. In addition, we found no association between neutralization and age, sex, or the Ct of the first positive RT-qPCR assessment (data not shown).

Finally, we observed that some sera seemed to enhance SARS-CoV-2pp infectivity. To explore whether antibodies could facilitate infection and hence virus spread, we tested the ADE effect mediated by sera from patients in the different groups (Supplemental Fig. 2), through infection of THP-1 cells that express different Fcγ receptors. We selected sera from those who developed mild (*n* = 7), moderate (*n* = 10), and severe (*n* = 9) forms of the disease, as well as sera from patients infected by other coronaviruses (*n* = 9). However, no increase was observed in any condition, suggesting that the severe forms of the disease observed in ICU patients were not ADE mediated.

Overall, these results indicate that nAb activity correlates highly with symptom severity, suggesting either that a robust humoral response is generated only in patients with severe symptoms or that the humoral response contributes to aggravation of the disease.

### SARS-CoV-2 anti-N and anti-S Abs correlate with the nAb response

**Fig. 2:**
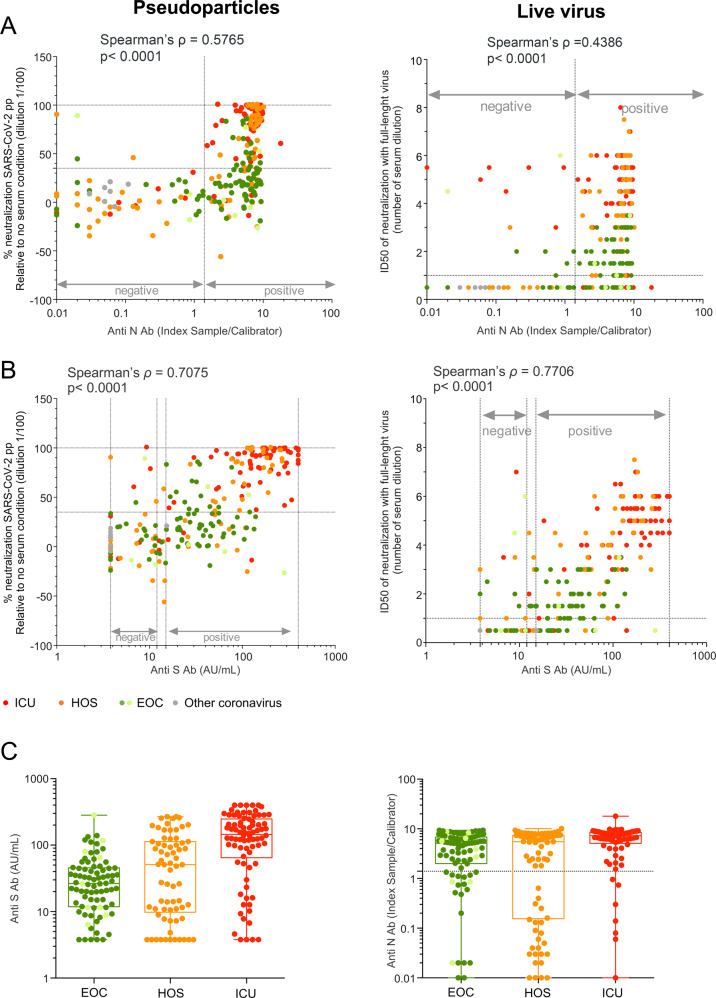
Serum neutralization of SARS-CoV-2 correlates with anti-S antibodies. **A** Correlation between the percentage of neutralization of SARS-CoV-2pp (left) or ID50 of live virus (right) with seroconversion measured by anti-N antibodies. **B** Same as **A** with anti-S antibodies. **C** Anti-S (left) and anti-N (right) IgG value distributions in the three groups of patients

The large number of EOC patients without detectable nAb activity raises the question of the identification of correlates of protection in infected patients, as high titers of nAbs are generally thought to confer protection against infection. Thus, we sought to identify whether IgG responses raised against components of SARS-CoV-2 particles correlate with the nAb activity detected in patient samples. To this end, anti-S and anti-N IgG responses were measured by two commercially available tests. We found that most samples from SARS-CoV-2-positive patients were positive in at least one assay (Fig. 2A, B). Interestingly, although anti-S IgG titers correlated highly with nAb titers (Spearman’s *ρ* = 0.7075, Fig. 2B), the correlation was lower between anti-N IgG and nAbs (Spearman’s *ρ* = 0.5765, Fig. 2A). Of note, anti-N and anti-S titers only showed a mild correlation (Spearman’s *ρ* = 0.5148, Supplemental Fig. 3). Such differences were particularly obvious when we compared the distribution of IgG among each category of patients. Indeed, although we found a low overlap in anti-S IgG profiles between EOC and ICU patients, which appeared similar to those of nAbs (compare Fig. 1C vs. Fig. 2C left), we detected a strong overlap in anti-N IgG values between the EOC and ICU patients (compare Fig. 1C vs. Fig. 2C right). This suggested that anti-S IgG values are relevant for marking the presence and levels of nAb activity. Indeed, our results indicate that 95% of sera with >124 AU/mL of anti-S IgGs could strongly neutralize SARS-CoV-2pp (>90% neutralization).

Overall, these results show that the anti-S IgG response can be used as a marker of neutralizing activity in individuals.

### SARS-CoV-2 Ab kinetics indicate a rapid waning of nAb activity in COVID-19 patients

**Fig. 3:**
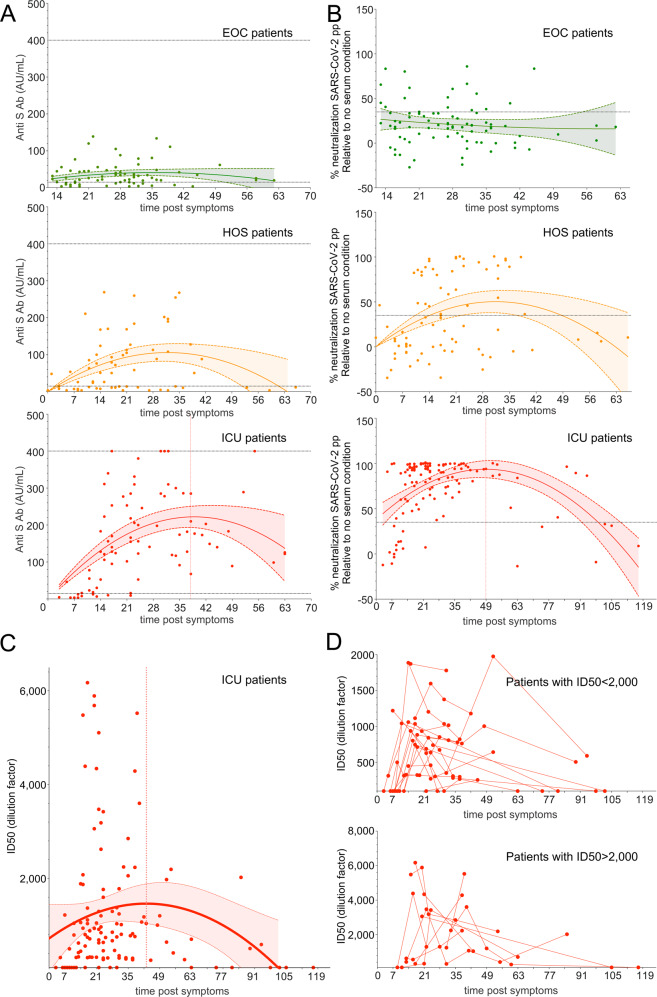
Appearance and longevity of neutralizing antibodies. **A** Seroconversion assessed by anti-S IgGs plotted against the days post-symptoms on which the samples were collected. **B** Percentage of neutralization of each serum sample assessed with SARS-CoV-2pp plotted against the days post-symptoms on which the samples were collected. The lines show the mean values expected from a second-order polynomial regression; the ribbons indicate the pointwise 95% confidence intervals. **C** Neutralizing antibody titers (ID50) assessed with SARS-CoV-2pp plotted against the days post-symptoms on which the samples were collected. The lines show the mean values expected from a second-order polynomial regression; the ribbons indicate the pointwise 95% confidence intervals. **D** Individual kinetics displayed for ICU patients with nAb titers (ID50) below (top) or above (bottom) 2000

Next, we evaluated the kinetics of anti-S IgG and nAb activities. Indeed, in addition to the diversity of clinical forms, one key feature of our cohort was serial serum sampling for most patients, allowing us to estimate the persistence of humoral factors. We found that both anti-S IgGs and nAb activities were generally detectable at 5–7 days post onset of symptoms in patients who developed a humoral response. These antibodies seemed to rapidly increase to reach a peak but to progressively decline from 40 days post onset (Fig. 3A, B). Moreover, the tendency for anti-S IgGs followed the same patterns as for nAbs, further confirming the correlation between anti-S IgG and neutralizing activity.

To gain further insight into the kinetics of nAb activity, we calculated the nAb titers (ID50) of each serum sample from ICU patients and found that the ID50 was relatively heterogeneous and high (2092 ± 1724), with values up to 6000 for some patients (Fig. 3C). These results also confirmed the rapid decline in neutralizing activity, as modeled with a second-order polynomial function that proved to be our best model among the different polynomials tested (Fig. 3C). Specifically, of 11 serum samples sampled after 60 days post onset, 7 patients showed no detectable neutralizing activity, and 4 patients had strongly reduced nAb titers with an over 4-fold drop in the ID50 median between the maximum and the last ID50 (median ID50_max_ = 2700, median ID50_final_ = 648.5) (Fig. 3D).

### No cross-neutralization of SARS-CoV-2 can be induced by previous infection with alternative coronaviruses

**Fig. 4:**
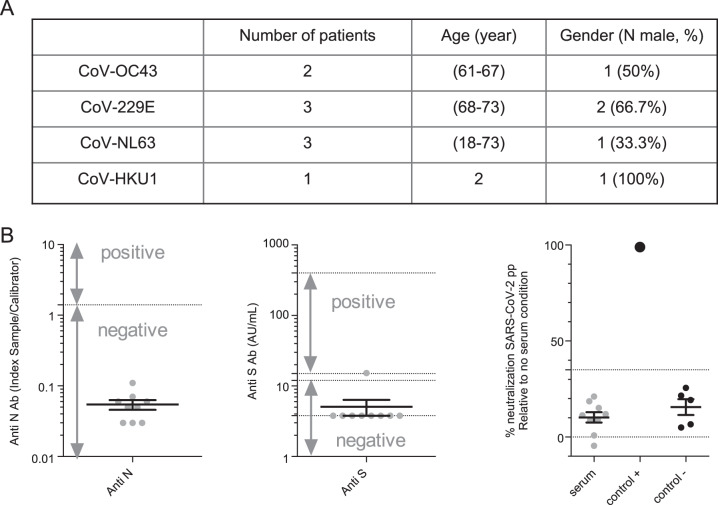
Sera from patients infected by endemic coronaviruses have no cross-neutralizing activity against SARS-CoV-2. **A** Characteristics of samples from patients infected with other coronaviruses. **B** Seroconversion assessed by anti-N (left) and anti-S (middle) SARS-CoV-2 or neutralization measured by SARS-CoV-2pp (right). For neutralization assays, a commercial anti-S antibody was used as a positive control (control+) and five prepandemic serum samples were used as a negative control (control−)

Previous reports have suggested that immunity for other coronaviruses may confer a certain degree of protection against endemic coronaviruses.^19^ Hence, we explored the possibility that serum specimens from individuals diagnosed with OC43, 229E, NL63, and HKU1 coronavirus infections (Fig. 4A) but not infected with SARS-CoV-2 could cross-neutralize SARS-CoV-2. However, we found that none of the tested samples showed neutralizing activity above the cutoff of detection (Fig. 4B), suggesting the absence of cross-neutralization between SARS-CoV-2 and endemic coronaviruses.

### D614G substitution is not associated with resistance to SARS-CoV-2 neutralization

**Fig. 5:**
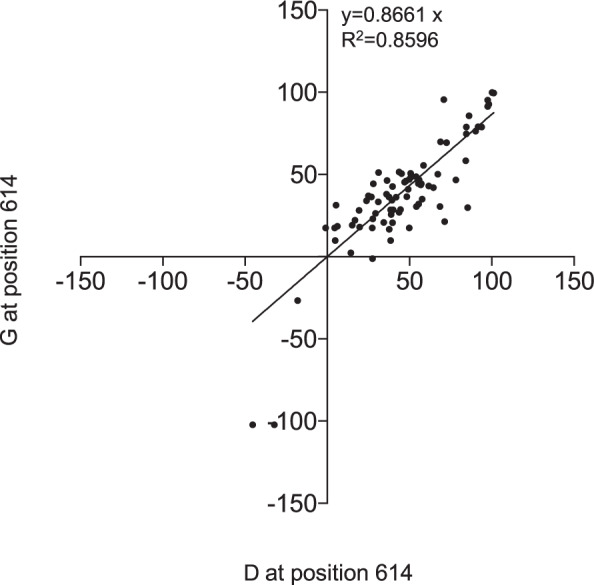
The residue at position 614 of SARS-CoV-2 spike does not influence the activity of nAbs. Percentage of neutralization of SARS-CoV-2pp using the spike protein with either a G or D at position 614

Considering the degree of mutations in coronaviruses, concerns have been raised about the emergence of SARS-CoV-2 immune escape mutants. In particular, the G614 spike protein variant has progressively emerged and replaced the D614 residue initially present in the Wuhan strain to become the dominant pandemic form, which currently constitutes >97% of the isolates worldwide.^13^ To address the possibility of a neutralization escape phenotype potentially conferred by the D614G mutation, we used the SARS-CoV-2pp assay, which is particularly suitable for comparing the nAb activity of serum specimens against pseudoparticles harboring this mutation. However, we found that the D614G mutation did not affect the nAb activity of the serum samples from our cohort, as shown by similar neutralization profiles (Fig. 5), indicating that this highly prevalent mutation does not play a role in SARS-CoV-2 neutralization escape but rather may modulate viral fitness and infection.^20^

## Discussion

Our understanding of the nature of the protective immune response to SARS-CoV-2 is currently limited but is likely to involve both cellular and humoral immunity. Here, we characterize some features of the humoral response in a cohort of individuals infected with SARS-CoV-2. The serum specimens used in this study were collected according to routine clinical management, without selection based on specific criteria. Although this may limit the statistical interpretation, this cohort represents a particularly appropriate picture of the different forms of severity among COVID-19 patients and thus allows an original longitudinal study.

Using either live virus or SARS-CoV-2pp assays, we compared the nAb response in sera from patients with different levels of COVID-19 severity according to their hospitalization status, including those in the ICU but also SARS-CoV-2-positive individuals with more moderate disease forms. Recent reports have evaluated the neutralizing activity of sera from COVID-19 patients and the results have been controversial. One study suggested that patients rapidly develop nAbs,^21^ whereas other studies have indicated that the humoral response^22^ and nAb activity^22–24^ correlate with several parameters, including the severity of the disease, resulting in the absence of nAb activity detection in a significant number of mild symptomatic and ASYs.^25^ However, such assumptions were based on a very limited number of observations, as those studies included less than 10 patients with the most severe forms. In contrast, our cohort is composed of 140 patients, one-third of whom were ICU patients with multiple sampling post onset. Those characteristics allowed us to address important questions, such as correlations of nAb activity with disease severity, IgG response, or kinetics of antibody levels. Here we confirm the heterogeneity of the humoral response in COVID-19 patients. Although we found that parameters, such as age or sex, were not associated with nAb activity, we show that among patients with the mildest disease forms, many did not exhibit robust neutralizing activities (Fig. 1). Importantly, we demonstrate that disease severity, as assessed by the hospitalization unit status, strongly correlated with levels of nAbs as well as with anti-S IgG titers (Figs. 1 and 2). This is in agreement with a recent observation that the strongest T-cell signals are found in patients with the most severe disease forms, raising the question of the beneficial vs. detrimental effect of T-cell activation in SARS-CoV-2 pathogenesis.^26^ Of note, as age is an important risk factor,^27^ most of the oldest patients showed both a severe form of the disease and high neutralizing activity, leading to possible confusion in identifying the main factors of the humoral response. Indeed, other studies^28^ observed a moderate correlation between age and neutralizing activity. Nevertheless, when we examined whether age is associated with nAb activity within the same disease severity group, we found no correlation, hence suggesting that disease severity is the main factor that explains neutralizing activity in patients. Finally, ADE has been proposed as a potential mechanism to explain the most severe cases of COVID-19.^29,30^ Regardless, no ADE was observed in our experimental setting, which may suggest that ADE is neither involved in the severe forms experienced by our patients nor provoked by previous infection by coronaviruses other than SARS-CoV-2.

Importantly, although for most viral infections, high nAb titers are usually associated with viral clearance, it seems that robust nAb activity does not confer protection against disease progression in COVID-19. As shown by a growing amount of clinical evidence,^31–33^ disease severity correlates with higher viral loads and hence with more antigens available to induce antibodies. However, in our cohort, no correlation between viral load and nAb activity was identified, even though the ICU patients presented a significantly higher initial viral load (Supplemental Fig. 4). Nonetheless, it should be noted that the sampling conditions of our patients may not have allowed us to accurately analyze a possible correlation. Indeed, the initial nasopharyngeal swabs used for the assessment of viral loads by RT-qPCR were obtained at different times post onset of infection (between 4 and 10 days in our case), depending on distinct patient clinical histories, and by different clinicians, which may lead to poorly reliable viral load assessments of the first qPCR-positive samples.^34^ Alternatively, a robust humoral response may be a feature of overall exaggerated immune activation in severe SARS-CoV-2 infection. In fact, antibodies mediate additional immune functions that may have both protective and pathological consequences. In addition, humoral responses in COVID-19 patients have been shown to correlate with cytokine and chemokine levels,^35^ which are the main effectors of severe systemic inflammatory responses known as “cytokine storms”, which are found in the most severe forms of the disease.^36^ Thus, an uncontrolled humoral response may also contribute directly to the pathogenesis of the disease by promoting organ damage, but this mechanism has yet to be demonstrated for SARS-CoV-2.

The correlation between seroconversion and nAb activity was also analyzed (Figs. 2 and 3). An initial report indicated that low IgG levels in serum are associated with severe forms of the disease,^37^ even though many recent publications have demonstrated the opposite.^38–40^ In addition, several studies by other groups^41,42^ have indicated that both anti-S and anti-N IgG levels correlate with nAb titers. In our study, both anti-S and anti-N IgG levels correlated with nAb titers; the correlation with anti-S antibodies was stronger, which likely reflects the variety of antibodies raised against SARS-CoV-2 determinants,^43^ with spike being the main target of nAbs for diverse coronaviruses, including SARS-CoV-2.^44^ Thus, if neutralizing activity is associated with protection against subsequent reinfections, the determination of anti-S IgG titers, by, e.g., enzyme-linked immunosorbent assay, may be useful for discriminating protected from unprotected individuals, in particular for patients with the most moderate forms, as they are less prone to develop robust neutralizing activity. In our cohort, 95% of the serum samples with anti-S antibody values above 124 AU/mL were associated with robust neutralization (90% neutralization or more), suggesting that anti-S antibody determination may be used as an evaluation of nAb activity rather than for anti-N IgG assays.

Due to the availability of several serial samples for most of the patients in the study, we also addressed the question of the stability of nAb levels. SARS-CoV-2 immunity appears to be protective in a rhesus macaque model and to persist for at least 35 days.^45^ However, the duration of such immunity in humans is still debated. We confirmed a tendency for a decrease in nAb activity and in anti-S IgG titers after reaching a peak, as previously shown by others,^46^ indicating that for some patients, nAb activity may be transient (Fig. 3). Importantly, for ICU patients, our results provide a more precise estimation of the persistence of nAb activity. Our models predict that SARS-CoV-2 nAb activity is likely to rapidly vanish and may not last for more than 4 months. Obviously, such mathematical models should be regarded carefully and will need to be re-evaluated when results using sera from late convalescent patients (>6 months) are obtained to better predict the decline or stability of nAb activity following recovery. Nevertheless, our results are in accordance with previous studies indicating a rapid decay in anti-SARS-CoV-2 antibodies in patients^40,47^ and with a recent report showing a loss in nAb activity that mirrored the reduction in antigen-specific IgA and IgM.^46^ Of note, relative stability of anti-RBD antibodies was observed in a cohort of 15 COVID-19 patients,^48–50^ which indicates that the kinetics of nAb activity in patients need to be further evaluated with alternative COVID-19 cohorts and more patients.

Overall, our results are in sharp contrast with SARS-CoV-induced disease, for which nAbs could be detected for ~270 days, with ID50 > 100.^51,52^ Thus, for SARS-CoV-2, it remains to be determined whether such waning of nAb activity is associated with an absence of protection or, alternatively, whether potential reinfections may trigger immune memory and induce faster and higher production of nAbs, eventually leading to better control of the infection and/or to reductions in symptoms. Further studies are warranted to evaluate the persistence of SARS-CoV-2 nAbs.

Naturally occurring variants in the S protein have been reported since the beginning of the pandemic. Among them, a variant with a single mutation at position 614 (D614G) has become the currently dominant circulating virus. This variant has been associated with increased infectivity, although it did not exhibit resistance to nAbs present in convalescent sera.^20^ By comparing the neutralizing activity for SARS-CoV-2pp with D614 and G614S, our report confirms that this mutation is not associated with resistance to neutralization.

Finally, we also analyzed nine serum samples from patients infected with alternative coronaviruses, which cause mild symptoms in adults, including respiratory illnesses and enteric and neurological diseases.^53^ It is important to understand cross-reactivity between other coronaviruses and SARS-CoV-2 immunity, as it might influence disease severity or response to a vaccine.^54^ In line with this, T-cell reactivity against SARS-CoV-2 was observed in unexposed people.^55^ Interestingly, by investigating the cross-reactivity of circulating Abs, we found that none of the nine samples displayed cross-reacting nAbs against SARS-CoV-2 infection. Furthermore, cross-neutralization of SARS-CoV-2 can be induced by sera from convalescent SARS-CoV patients, which is likely due to high homology between these two viruses, though it seems to be serum dependent.^56,57^ Thus, our results indicate an absence of cross-neutralization between SARS-CoV-2 and other endemic human coronaviruses, suggesting that nAbs or other non-nAbs^6^ generated by previous infections with other coronaviruses do not protect against SARS-CoV-2 infection.

## Supplementary information

Supplemental figures 1 to 4
