# Supplementary material for: A longitudinal study of SARS-CoV-2-infected patients reveals a high correlation between neutralizing antibodies and COVID-19 severity
Source: Cell Mol Immunol. 2021 Jan 6;18(2):318–27. doi: 10.1038/s41423-020-00588-2 (PMC7786875; doi:10.1038/s41423-020-00588-2)
Supplement: Supplementary file 1 — Supplemental figures 1 to 4 [file 41423_2020_588_MOESM1_ESM.pdf]

A longitudinal study of SARS-CoV-2 infected patients shows high correlation between neutralizing antibodies and COVID-19 severity

Short title

Neutralizing antibodies and COVID-19 severity

Authors

Vincent Legros[1,2,*], Solène Denolly[1,*], Manon Vogrig[3,4], Bertrand Boson[1], Eglantine Siret[1], Josselin Rigaill[3,4], Sylvie Pillet[3,5], Florence Grattard[3,5], Sylvie Gonzalo[3], Paul Verhoeven[3,5], Omran Allatif[1], Philippe Berthelot[5,6], Carole Pélissier[7], Guillaume Thiery[8], Elisabeth Botelho-Nevers[5,6], Guillaume Millet[9], Jérôme Morel[10], Stéphane Paul[4,5], Thierry Walzer[1], François-Loïc Cosset[1,$,**], Thomas Bourlet[3,5,**] and Bruno Pozzetto[3,5,**]

Affiliations

[1]CIRI – Centre International de Recherche en Infectiologie, Team EVIR, Univ Lyon, Université Claude Bernard Lyon 1, Inserm, U1111, CNRS, UMR5308, ENS Lyon, 46 allée d'Italie, F-69007, Lyon, France.

[2]Université de Lyon, VetAgro Sup, Marcy-l'Étoile, France.

[3]Department of Infectious Agents and Hygiene, University-Hospital of Saint-Etienne, Saint-Etienne, France

[4]Department of Immunology, University-Hospital of Saint-Etienne, Saint-Etienne, France

[5]CIRI – Centre International de Recherche en Infectiologie, Team GIMAP, Univ Lyon, Université Claude Bernard Lyon 1, Inserm, U1111, CNRS, UMR5308, ENS Lyon, 46 allée d'Italie, F-69007, Lyon, France.

[6]Department of Infectious Diseases, University-Hospital of Saint-Etienne, Saint-Etienne, France.

[7]Department of Occupational Medicine, University-Hospital of Saint-Etienne, Saint-Etienne, France.

[8]Department of Intensive Care and Resuscitation (Réanimation G), University-Hospital of Saint-Etienne, Saint-Etienne, France

[9]Laboratoire Interuniversitaire de Biologie de la Motricité, Université de Lyon, Université Jean Monnet, Saint-Etienne, France.

[10]Department of Anesthesiology and Critical Care, University-Hospital of Saint-Etienne, Saint-Etienne, France.

*These two authors contributed equally to this work.

**These three authors contributed equally to this work.

[$]Corresponding author: Address: ENS Lyon, 46 allée d'Italie, F-69007, Lyon, France. tel: +33472728732; e-mail: flcosset@ens-lyon.fr

Funding

The laboratory of FLC received financial support from the LabEx Ecofect (ANR-11-LABX-0048) of the ‘‘Université de Lyon”, within the program ‘‘Investissements d'Avenir” (ANR-11-IDEX-0007) operated by the French National Research Agency (ANR), the ANR (grant from RA-Covid-19), the Fondation pour la Recherche Médicale (FRM), and Inserm Transfert.

Conflict of Interest disclosure statement

The authors have declared that no competing interests exist.

Supplemental Figure Legends

Supplemental Figure 1. Serum neutralization of RD-114pp.

Percentage of neutralization with RD-114pp for each patient classified according to the unit of hospitalization. For patients with serial serum samples, the sera collected at the closest to twenty days post-onset of symptoms were chosen.

Supplemental Figure 2. Absence of antibody-dependent enhancement in patients' sera.

Percentage of infection obtained with SARS-CoV-2pp and VSV-Gpp in THP-1 cells. Pseudoparticles were pre-incubated with sera from patients classified according to the hospitalization status (ICU, HOS, EOC) or infected with a non-SARS-CoV-2 coronavirus (CoV). The sera were diluted at 1/100 (**A**) or 1/5,000 (**B**).

Supplemental Figure 3. Correlation between anti-N and anti-S antibodies.

The values of seroconversion measured by anti-N and anti-S antibodies are shown for the different groups of patients.

Supplemental Figure 4. Viral loads in firstly collected samples correlate with disease severity.

Ct values of the first RT-qPCR (from samples obtained between day 4 and day 10 post-onset of infection) for each patient classified according to the hospitalization status.

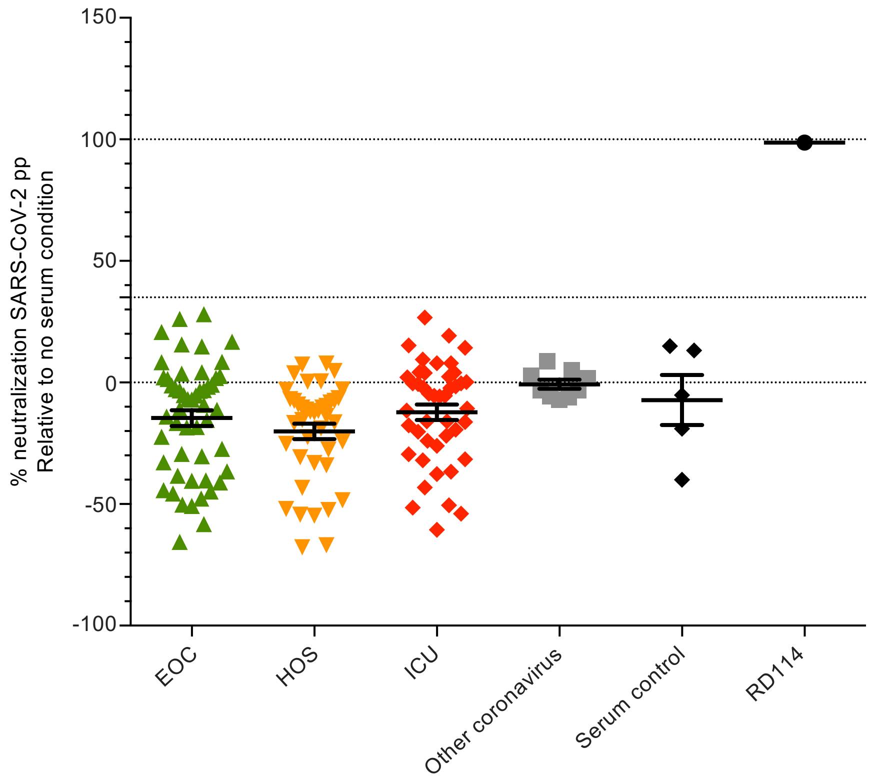

Supplemental Figure 1

A

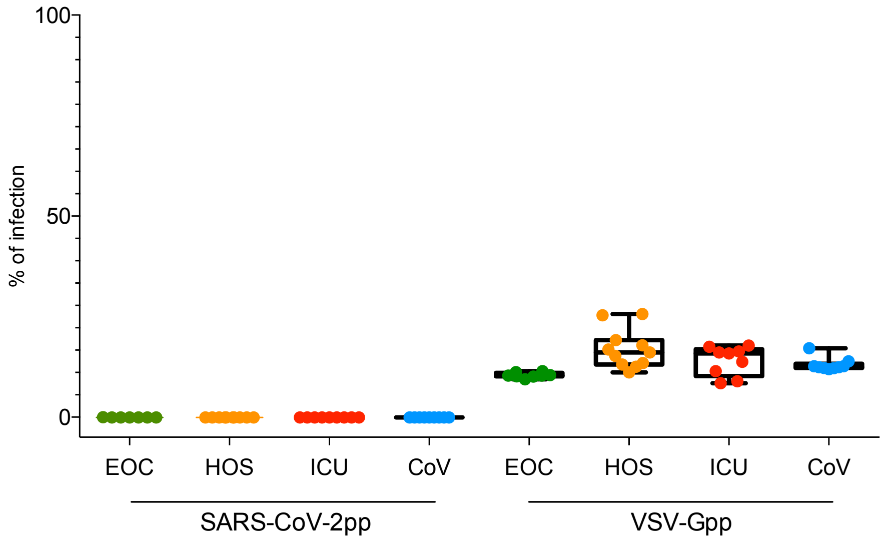

B

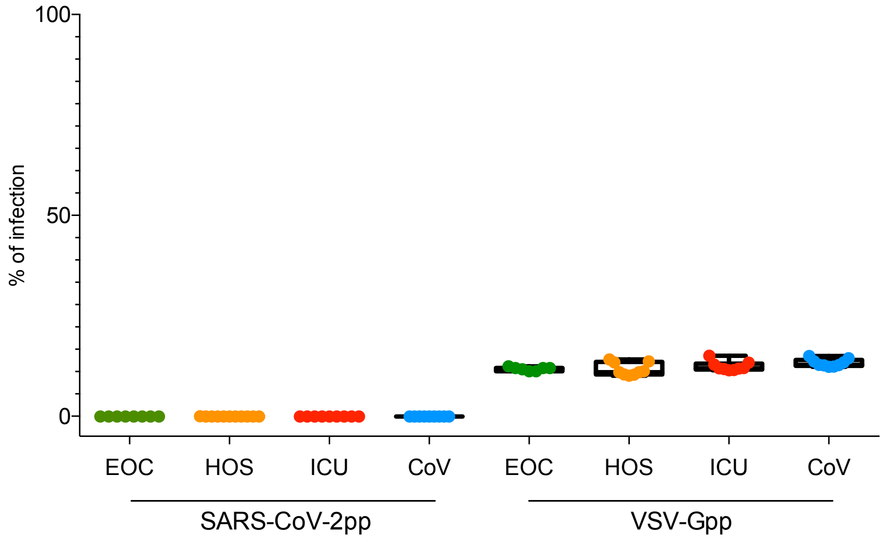

Supplemental Figure 2

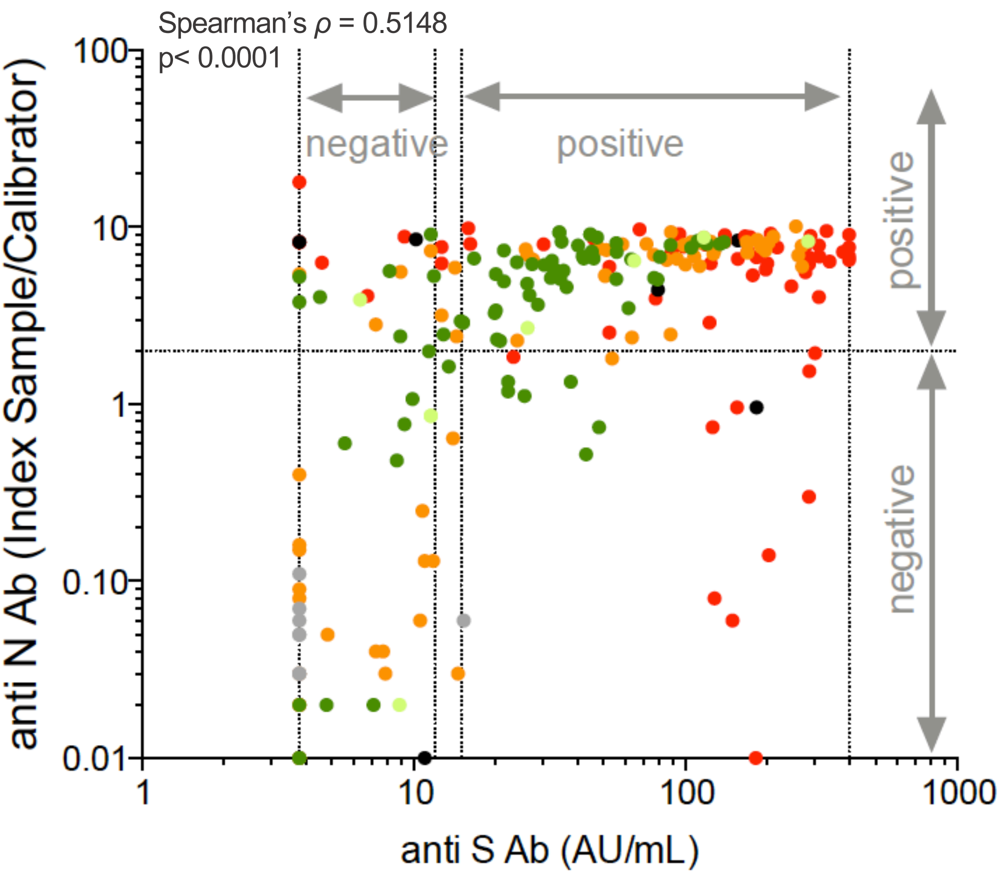

Supplemental Figure 3

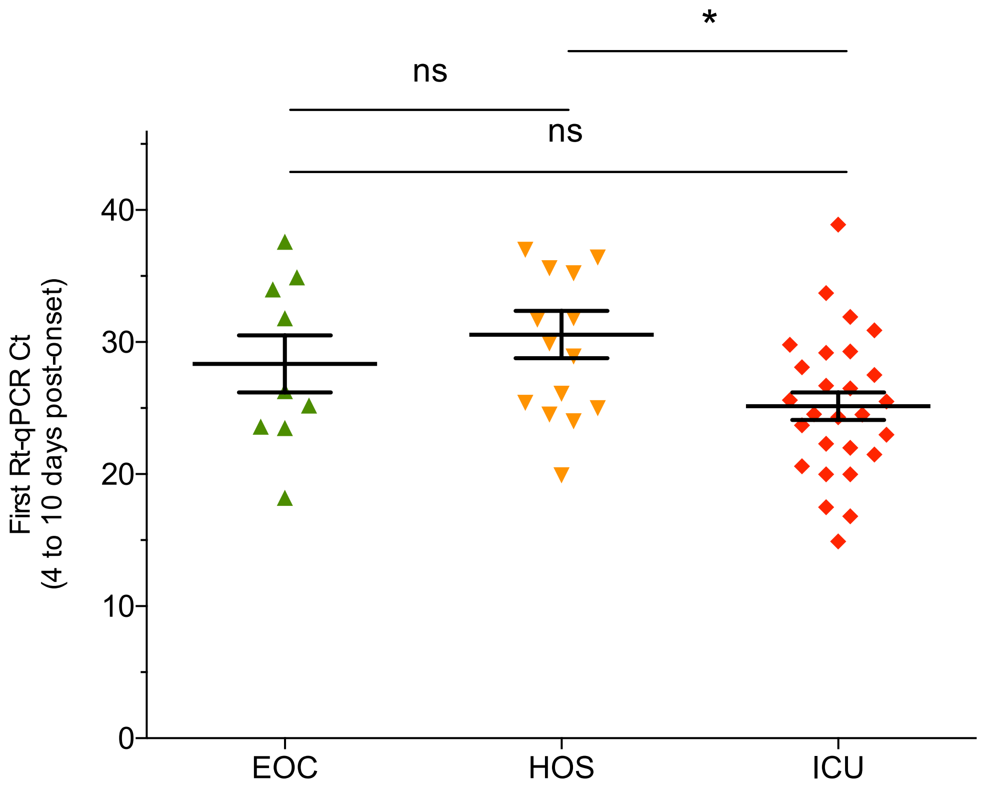

Supplemental Figure 4
